# Influence of seasonal exposure to grass pollen on local and peripheral blood IgE repertoires in patients with allergic rhinitis

**DOI:** 10.1016/j.jaci.2014.07.010

**Published:** 2014-09

**Authors:** Yu-Chang B. Wu, Louisa K. James, Jason A. Vander Heiden, Mohamed Uduman, Stephen R. Durham, Steven H. Kleinstein, David Kipling, Hannah J. Gould

**Affiliations:** a Randall Division of Cell and Molecular Biophysics, King's College London, London, United Kingdom; b Medical Research Council and Asthma UK Centre, Allergic Mechanisms in Asthma, London, United Kingdom; c Interdepartmental Program in Computational Biology and Bioinformatics, Yale University, New Haven, Conn; d Department of Pathology, Yale School of Medicine, New Haven, Conn; e Allergy and Clinical Immunology, National Heart and Lung Institute, Imperial College London, London, United Kingdom; f Institute of Cancer & Genetics, School of Medicine, Cardiff University, Cardiff, United Kingdom

**Keywords:** Next-generation sequencing, peripheral blood and nasal mucosal IgE repertoires, allergic rhinitis, AR, Allergic rhinitis, AR.IS, Allergic rhinitis inside the pollen season, AR.OS, Allergic rhinitis outside the pollen season, CDR, Complementarity-determining region, CSR, Class-switch recombination, GC, Germinal center, NA, Nonallergic healthy control subject, NGS, Next-generation sequencing, QC, Quality control, SHM, Somatic hypermutation

## Abstract

**Background:**

Previous studies of immunoglobulin gene sequences in patients with allergic diseases using low-throughput Sanger sequencing have limited the analytic depth for characterization of IgE repertoires.

**Objectives:**

We used a high-throughput, next-generation sequencing approach to characterize immunoglobulin heavy-chain gene *(IGH)* repertoires in patients with seasonal allergic rhinitis (AR) with the aim of better understanding the underlying disease mechanisms.

**Methods:**

*IGH* sequences in matched peripheral blood and nasal biopsy specimens from nonallergic healthy control subjects (n = 3) and patients with grass pollen–related AR taken in season (n = 3) or out of season (n = 4) were amplified and pyrosequenced on the 454 GS FLX+ System.

**Results:**

A total of 97,610 *IGH* (including 8,135 IgE) sequences were analyzed. Use of immunoglobulin heavy-chain variable region gene families 1 *(IGHV1)* and 5 *(IGHV5)* was higher in IgE clonotypic repertoires compared with other antibody classes independent of atopic status. IgE repertoires measured inside the grass pollen season were more diverse and more mutated (particularly in the biopsy specimens) and had more evidence of antigen-driven selection compared with those taken outside of the pollen season or from healthy control subjects. Clonal relatedness was observed for IgE between the blood and nasal biopsy specimens. Furthermore in patients with AR, but not healthy control subjects, we found clonal relatedness between IgE and IgG classes.

**Conclusion:**

This is the first report that exploits next-generation sequencing to determine local and peripheral blood *IGH* repertoires in patients with respiratory allergic disease. We demonstrate that natural pollen exposure was associated with changes in IgE repertoires that were suggestive of ongoing germinal center reactions. Furthermore, these changes were more often apparent in nasal biopsy specimens compared with peripheral blood and in patients with AR compared with healthy control subjects.

The immunoglobulin repertoire in the periphery is shaped by somatic hypermutation (SHM), class-switch recombination (CSR), and affinity maturation within the germinal centers (GCs) of lymphoid tissues in response to antigens. The pivotal role of IgE in allergic inflammation is well characterized,^1^ particularly as highlighted by the clinical efficacy of anti-IgE therapy.^2^ Recently, several mouse models have been developed in an attempt to unravel the mystery of IgE^+^ B-cell ontogeny^3^; however, mechanisms behind the prevalence of IgE^+^ B cells in human subjects remain unclear. Although Sanger sequencing has been applied to study IgE repertoires in patients with allergic disease,^4^, ^5^ its low sequence yield and limited coverage has resulted in conflicted findings, such as the role of classical T cell–dependent antigens versus superantigens in shaping the selected IgE repertoire in patients with allergic disease.^6^, ^7^, ^8^, ^9^, ^10^, ^11^, ^12^

Over recent years, sequencing technologies have evolved dramatically, advancing from the low-throughput Sanger-based methods to massively parallel and high-throughput approaches that are enabled by several next-generation sequencing (NGS) platforms.^13^ In 2009, Weinstein et al^14^ published the first NGS study on zebrafish immunoglobulin repertoires, and just months later, Boyd et al^15^ demonstrated the feasibility of NGS for monitoring immunoglobulin repertoires in clinical specimens. Since then, NGS technologies have been applied to various studies of B-cell development,^16^, ^17^, ^18^, ^19^, ^20^ vaccination responses,^21^, ^22^ cancer,^23^ and both infectious^24^, ^25^ and autoimmune diseases.^26^ The unprecedentedly large numbers of immunoglobulin sequences generated by using NGS technologies have revolutionized our ability to determine the abundance, relatedness selection, and SHM of B-cell clones, thus providing a wealth of information relevant to the generation of immune memory and antibody responses in both health and disease. Applications of NGS technologies have expanded beyond basic immunology research into drug discovery^27^, ^28^ and clinical diagnostics.^15^, ^29^ In combination with mAb expression and structural biology, NGS technologies have led to the discovery of broadly neutralizing antibodies against HIV-1.^25^, ^28^ The potential value of NGS for clinical biomarker discovery has also been explored.^21^, ^23^, ^30^ Despite this, NGS technologies have not yet been applied to the study of IgE repertoires in patients with respiratory allergic disease.

Seasonal allergic rhinitis (AR) affects a quarter of the population of westernized countries, and a large proportion of these patients are allergic to pollens. In this report a high-throughput NGS approach is introduced to characterize immunoglobulin heavy-chain gene *(IGH)* repertories in matched peripheral blood and nasal mucosal biopsy specimens from patients with AR inside the grass pollen season (AR.IS group), patients with AR outside the pollen season (AR.OS group), and nonallergic healthy control subjects (NA group). We detected significant changes in the IgE repertoire (as well as those of other antibody classes) in the AR.IS group with evidence of enhanced affinity maturation for IgE as a result of natural exposure to seasonal grass pollen. This report demonstrated the technical feasibility and usefulness of high-throughput NGS repertoire analysis in respiratory allergic disease research.

## Methods

### Study participants

Subjects with different atopic statuses, the AR.OS group (n = 3), the AR.IS group (n = 4), and the NA group (n = 3), were recruited from the Royal Brompton Hospital London allergy clinic or through local advertisement (see the Methods section and Table E1 in this article's Online Repository at www.jacionline.org). Samples were collected after obtaining written informed consent, as approved by the East London & The City REC Alpha (09/H0704/67).

### Sample processing

Nasal biopsy specimens (2.5 mm) were taken from the inferior turbinate after achievement of local anesthesia and subsequently homogenized with a Qiagen TissueLyser (Qiagen, Hilden, Germany). Peripheral blood lymphocytes were isolated from venous blood by using Ficoll density gradient separation (GE Healthcare, Fairfield, Conn). Total RNA was extracted with the RNeasy Mini Kit (Qiagen), and cDNA was synthesized by using SuperScript III RT (Invitrogen, Carlsbad, Calif).

### 454 Pyrosequencing of *IGH* libraries

As previously described,^21^ libraries containing *IGH* sequences were generated by means of seminested PCR reactions (see the Methods section and Table E2 in this article's Online Repository at www.jacionline.org) with a mixture of sense primers (framework region 1/immunoglobulin heavy-chain variable region *[IGHV]* gene families 1-7 for respective framework 1 regions) in conjunction with antisense primers (IGα, IGγ, IGε, and IGμ for IgA, IgG, IgE, and IgM, respectively). Processed library *IGH* sequences were pyrosequenced on the 454 GS FLX+ System (Roche, Mannheim, Germany).

### Sequence analysis pipeline

As previously described,^21^ the analysis pipeline has 4 components: an initial quality control (QC), IMGT/HighV-QUEST annotation, hierarchic clonotype clustering, and designation of clonotypic sequences (see the Methods section in this article's Online Repository). For some analyses, sequences were clustered by using more stringent criteria (see the Methods section in this article's Online Repository).

### Analysis of selection strength and clonal diversity

Selection strength for complementarity-determining regions (CDRs) and framework regions in sampled immunoglobulin sequences was estimated by using BASELINe (see the Methods section in this article's Online Repository).^31^ Clonal diversity was analyzed by using the model proposed by Hill (see the Methods section in this article's Online Repository).^32^

### Construction of lineage trees

The Phylogeny Inference Package (PHYLIP)^33^ was used to construct lineage trees containing unique clonal members with sequence variations. Sequences were further aligned against germlines where necessary by using the Lasergene Genomics Suite (DNAstar, Madison, Wis) for validation of their clonal relatedness.

### Statistics

Depending on the nature of data sets, different statistical methods were used for multiple group comparisons by using GraphPad Prism 6.0 software (GraphPad Software, La Jolla, Calif; see the Methods section in this article's Online Repository). Metrics of association were determined by using Pearson correlation and linear regression.

## Results

### Filtering and clonotype clustering of *IGH* sequences

A total of 152,784 sequence reads were generated from 20 samples. After QC analysis, 97,610 *IGH* sequences were identified, comprising 8,135 full-length IgE sequences (see Table E3 in this article's Online Repository at www.jacionline.org). QC-filtered sequences can be searched on the National Center for Biotechnology Information's Sequence Read Archive (Sequence Read Archive study accession no. SRP038092, see Table E1). Hierarchic clustering then allowed us to group clonally related sequences based on the third *IGH* CDR (CDR-H3) DNA motifs and identify 35,175 clonotypic sequences to represent their clonal families (see Table E4 in this article's Online Repository at www.jacionline.org).

### Abundance of *IGH* segments in clonotypic repertoires

**Fig 1:**
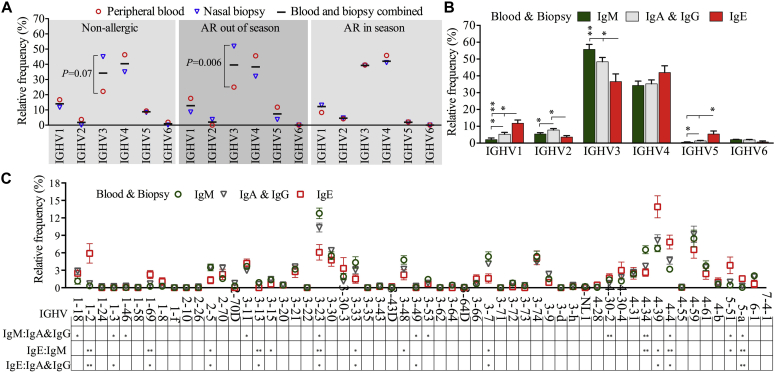
Abundance of *IGHV* genes in clonotypic repertoires by antibody class and sample type. **A** and **B,** Use of *IGHV* gene families in IgE clonotypic repertoires was compared across sample types and patient groups (Fig 1, *A*) or with other antibody classes (Fig 1, *B*). **C,** Use of individual *IGHV* genes was compared between antibody classes. *P < .05 and **P < .005. Fig 1, *B* and *C*, Groups of clonotypic sequences were combined from peripheral blood and biopsy specimens independent of atopic status.

Specific antigen challenge might enrich B cells expressing particular immunoglobulin genes, changing the landscape of immunoglobulin repertoires.^16^ For example, *IGHV5* genes have been associated with antibody responses to superantigens. Therefore we compared the relative abundance of immunoglobulin genes to determine whether natural pollen exposure increased particular *IGH* gene rearrangements. We observed that the use of *IGHV* gene families (Fig 1, *A*), *IGHD* gene families (see Fig E1, *A*, in this article's Online Repository at www.jacionline.org), and *IGHJ* gene families (see Fig E1, *B*) for IgE clonotypes did not significantly differ between allergic and nonallergic subjects. Differences between peripheral blood and biopsy specimens were very minor. However, we detected increased use of *IGHV3* gene families and a trend for reduced use of *IGHV4* gene families in nasal biopsy specimens from the NA and AR.OS groups but not the AR.IS group compared with their counterparts in peripheral blood.

Similar patterns of *IGH* gene rearrangements between IgA and IgG repertoires allowed us to group them as one switched IgE^−^ (IgA and IgG) population.^16^, ^17^ IgE sequences as a whole (IgE sequences from all 20 samples combined) showed a significant increase in the use of *IGHV1* and *IGHV5* gene families but a reduction for *IGHV2* and *IGHV3* (Fig 1, *B*) compared with IgM and switched IgE^−^ sequences. Similar patterns were observed separately in peripheral blood (see Fig E1, *C*) and nasal biopsy specimens (see Fig E1, *D*). IgE repertoires were also distinct significantly from other classes in their use of *IGHD* gene families (see Fig E1, *E*), *IGHJ* gene families (see Fig E1, *F*), and individual *IGHV* genes (Fig 1, *C*). Approximately 11 of 58 *IGHV* genes, such as *IGHV5-a*, differed in abundance for IgE clonotypic repertoires compared with other classes. In addition, significant changes between patient groups were observed in the use of some *IGHV* families (*IGHV1*, *IGHV2*, and *IGHV5*) for IgM (see Fig E1, *G* and *H*) and switched IgE^−^ (see Fig E1, *I* and *J*) repertoires.

### *IGHV* mutation status

**Fig 2:**
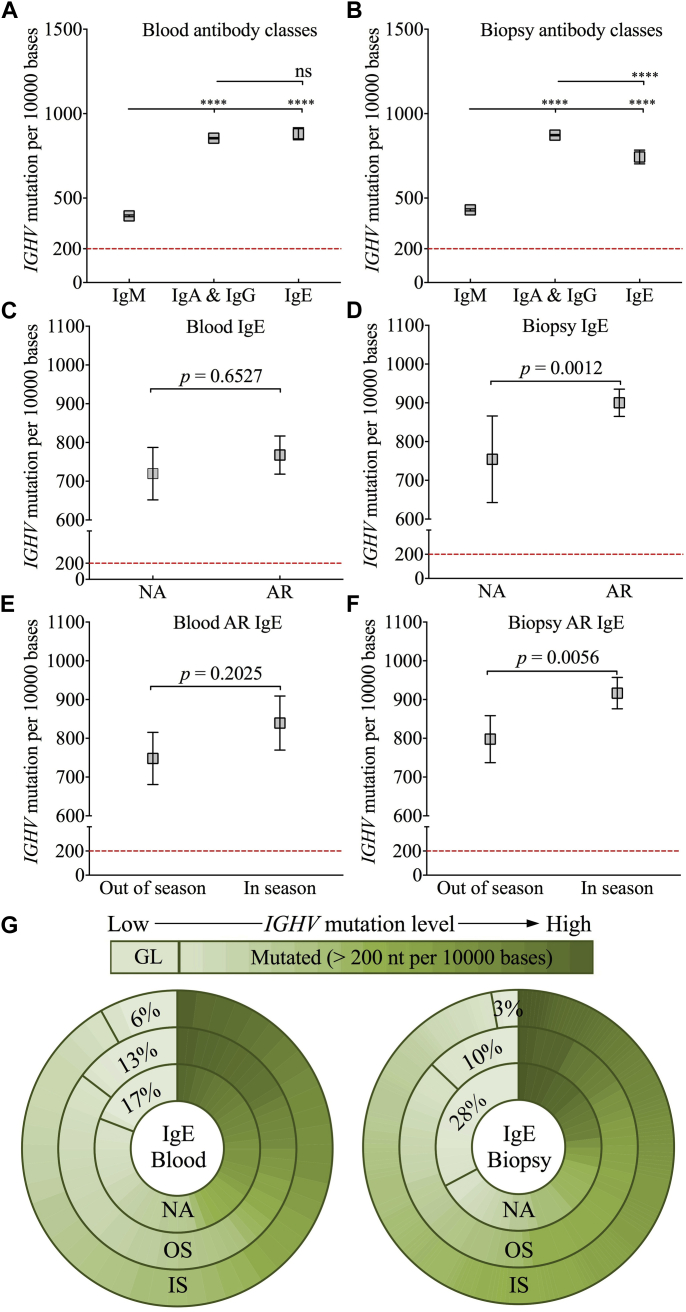
Changes in *IGHV* mutation levels by antibody class and sample type. **A** and **B,** Mean mutation frequencies were compared between clonotypic sequences of different antibody classes in peripheral blood (Fig 2, *A*) and nasal biopsy specimens (Fig 2, *B*). IgA and IgG repertories were combined as one switched IgE^−^ population. **C-F,** Mean mutation levels in IgE sequences were further compared between the NA and AR groups (Fig 2, *C* and *D*) or between groups of sequences taken in and out of season (Fig 2, *E* and *F*). *Red line*, Two percent background error rate. **G,** Pie charts show relative fractions of IgE clonotypes ranked by mutations in peripheral blood and nasal biopsy specimens from the NA group *(inner circles)*, AR.OS group *(middle circles)*, and AR.IS group *(outer circles)*. *GL*, Germline like, ≤200 mutations per 10,000 bases; *Mutated*, >200 mutations per 10,000 bases. ****P < .00005. *ns*, Not significant.

Increased mutation numbers in the *IGHV* region are indicative of higher-affinity antigen binding.^34^ Therefore the frequency of *IGHV* mutations was determined as the number of nucleotide differences compared with germline sequence per 10,000 bases. IgE and switched IgE^−^ sequences had significantly more mutations than IgM sequences (Fig 2, *A* and *B*). Within the IgE class, there were more mutations in the AR group (Fig 2, *C* and *D*), with further increases observed in biopsy specimens taken in season (Fig 2, *E* and *F*). Regardless of antibody class, the mutation frequency was increased (Fig 2, *D*, *F*, and *G*), and the proportion of germline-like (≤ 200 mutations per 10,000 bases) sequences was significantly reduced in biopsy specimens taken in season (see Fig E2, *A* and *B*, in this article's Online Repository at www.jacionline.org). In contrast, the opposite pattern for IgM and switched IgE^−^ sequences was observed in peripheral blood.

We further analyzed the degree of sequence variations among related mutants within each IgE clonal family as a way of assessing intraclonal diversification as a cause of clonal expansion and SHM. Thus intraclonal variation was calculated as the SD of *IGHV* mutations among all clonally related sequences. IgE clones taken in season had the highest degree of intraclonal variations for larger clones (clone size >3 sequences per clone; see Fig E2, *C*) and were more mutated compared with those taken out of season (see Fig E2, *D*).

### Physicochemical properties of CDR-H3

**Fig 3:**
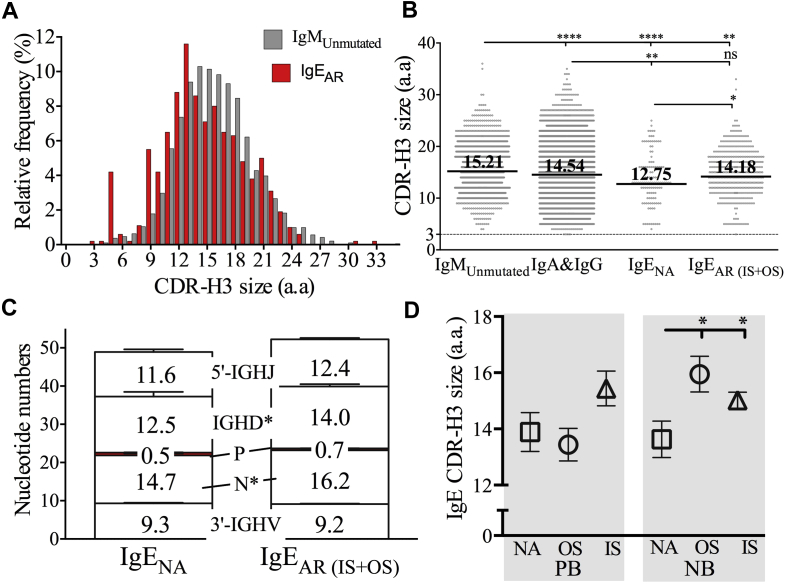
Patterns of CDR-H3 length. **A,** Virtual CDR-H3 spectratypes show the relative distribution of clonotypic sequences by CDR-H3 length for unmutated IgM *(gray bars)* and IgE_AR_ (*red bars*; AR.IS and AR.OS groups combined). *a.a*, Amino acids. **B,** Mean CDR-H3 length was compared between groups, as indicated. **C,** Nucleotide numbers for different CDR-H3 components in IgE sequences were compared between the IgE_NA_ and IgE_AR_ groups (AR.IS and AR.OS groups combined). **D,** Mean CDR-H3 length for IgE clonotypic sequences was compared between groups in peripheral blood *(PB)* and nasal biopsy specimens *(NB)*. *P < .05, **P < .005, and ****P < .00005. *ns*, Not significant.

CDR-H3 is regarded as the most important motif within immunoglobulin genes because of its central position in the antigen-binding pocket. Therefore we analyzed the physiochemical properties of CDR-H3 peptides. CDR-H3 motifs of unmutated (100% germline) IgM sequences were extracted from the whole data set to represent the most diverse fraction for comparison. Among antibody classes, CDR-H3 was longest in length for IgM but shortest for IgE (Fig 3, *A* and *B*). IgE sequences from allergic subjects (in and out of season combined) had significantly longer CDR-H3 (60% < 15 nucleotides; see Fig E3, *A*, in this article's Online Repository at www.jacionline.org) than the NA group (80% <15 nucleotides). This was mainly due to increased N-nucleotide numbers and *IGHD* lengths in allergic subjects (Fig 3, *C*). This pattern remained evident for IgE isolated from peripheral blood, as well as IgE from biopsy specimens (Fig 3, *D*). Although the CDR-H3 length for IgE did not differ in allergic subjects between samples taken in or out of season (data not shown), we observed different patterns in IgM and switched IgE^−^ repertoires between different atopic status (see Fig E3, *B* and *C*).

The number of *IGHV* mutations within an immunoglobulin sequence often negatively correlates with its CDR-H3 length.^35^, ^36^ In keeping with this, we observed a negative correlation between CDR-H3 lengths and *IGHV* mutations for IgE from the NA group (see Fig E4, *A*, in this article's Online Repository at www.jacionline.org) and AR.OS group (see Fig E4, *B*) samples. However, this correlation was not observed in the AR.IS group (Fig E4, *C*). In addition, we did not detect any significant differences of CDR-H3 peptide properties, including the aliphatic index, grand average of hydropathicity index, and theoretic isoelectric point (see Fig E5 in this article's Online Repository at www.jacionline.org), when comparing IgE sequences between the patient groups.

### Analysis of selection strength and clonal diversity

**Fig 4:**
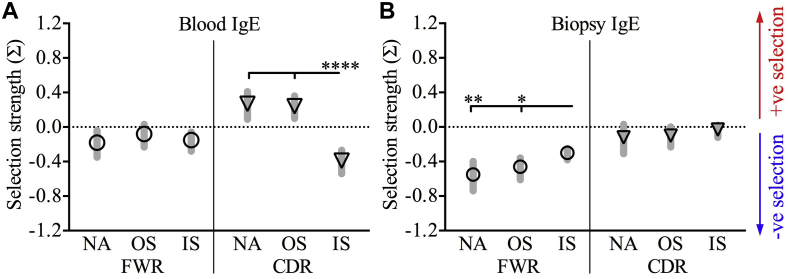
Selection strength of IgE sequences. Mean selection strength *(Σ)* for CDRs and framework regions *(FWRs)* for IgE clones in peripheral blood **(A)** or nasal biopsy specimens **(B)** were compared between atopic statuses. Values indicate means ± 95% CIs. *P < .05, **P < .005, and ****P < .00005.

B-cell selection is a pivotal process for affinity maturation of antibodies in which positive selection can often improve antibody affinity and negative selection can contribute to the structural integrity of antibodies.^31^ Therefore we used BASELINe to estimate selection strength imposed on the *IGH* repertoires. When comparing IgE sequences between the AR.IS and AR.OS groups, we observed distinctive patterns of selection (Fig 4). For IgE sequences from the AR.IS group, we found evidence of stronger negative selection in peripheral blood (Fig 4, *A*); however, nasal biopsy specimens showed stronger positive selection (Fig 4, *B*) compared with other atopic statuses.

**Fig 5:**
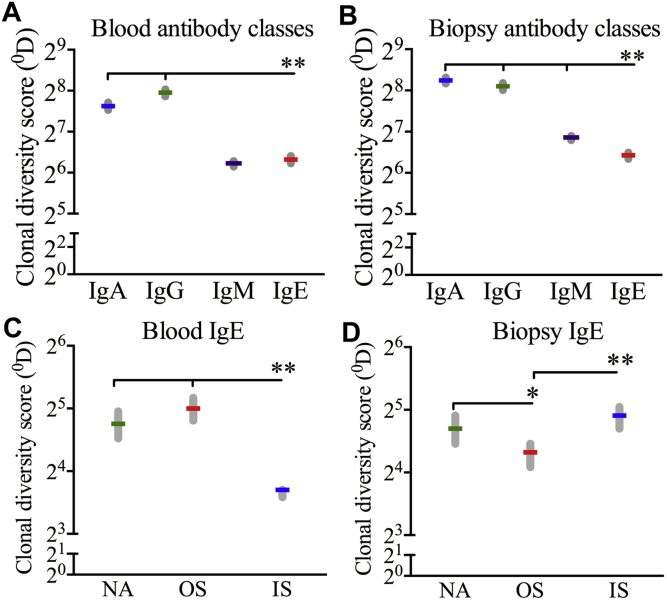
Analysis of clonal diversity using Hill's model.^32^ The diversity of total clones *(*^*0*^*D)* was compared across antibody classes in peripheral blood **(A)** and nasal biopsy specimens **(B)** or between IgE clones from different patient groups in peripheral blood **(C)** and nasal biopsy specimens **(D)**. Values indicate means ± 95% CIs. *P < .05 and **P < .005.

The repertoire diversity of B cells is influenced by selection strength, and a repertoire with a higher degree of diversity is more likely to recognize a wider array of antigens (ie, a less focused immune response) than a more restricted repertoire. To quantify diversity (^0^D) within each population of clones, we used the generalized diversity model proposed by Hill (see the Methods section and Fig E6 in this article's Online Repository at www.jacionline.org).^32^ IgE clones were the least diverse compared with other antibody classes (Fig 5, *A* and *B*).^32^ Within the IgE class, IgE clones from peripheral blood taken in season were the least diverse (Fig 5, *C*), whereas IgE clones from nasal biopsy specimens taken in season had the highest measure of diversity (Fig 5, *D*).

### IgE lineage trees and clonal relatedness

**Fig 6:**
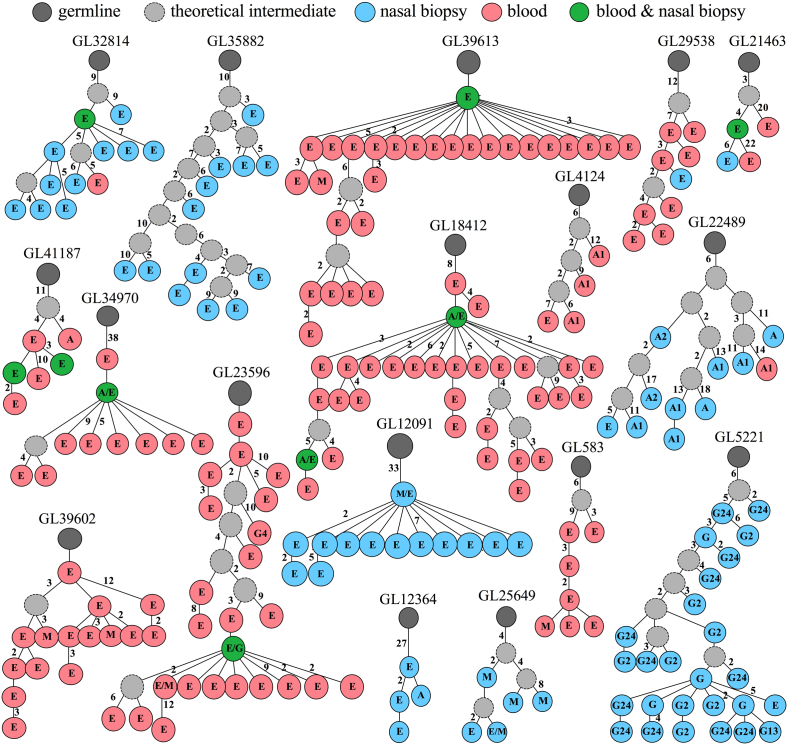
Examples of lineage trees containing IgE sequences. Only representative trees are presented for illustration purposes. Sequences from peripheral blood or nasal biopsy specimens are indicated by *colored circles*, with the number of point mutations between 2 adjacent sequences shown next to each branch (point mutation = 1 when not indicated). Antibody classes (*A*, IgA; *G*, IgG; *E*, IgE; *M*, IgM) and subclasses (*G1-G4*, IgG; *A1* and *A2*, IgA) are indicated within the *circles*. Only unique sequences with identifiable antibody classes are shown. Detailed information for the 12 trees containing IgE sequences and their related variants of other classes are shown in Table E6.

Lineage tree analysis was used to identify sequences across different antibody classes, sample types, or both. By clustering clonally related sequences and analyzing their mutation patterns, we were able to generate 1,752 lineage trees (data not shown). Of these, IgE sequences were present in 146 lineage trees (see Table E5). Trees containing only IgE sequences (eg, GL35882; Fig 6) or IgE and their variants of other antibody classes (eg, GL12091) were detected in nasal biopsy specimens. Twenty-one trees were associated with IgE sequences in peripheral blood to their IgE relatives in nasal biopsy specimens without a clearly defined lineage order (eg, GL32184, GL21643, and GL41187). Twelve lineage trees displayed molecular footprints that linked IgE to other classes (see Table E6). The relative frequencies of trees containing IgE sequences related to IgM (eg, GL12091 and GL25649) or IgE sequences related to IgA (eg, GL18412 and GL22489) did not significantly differ between allergic subjects and nonallergic subjects (Table I). In contrast, we found clonal relatedness between IgE and IgG classes in 2 of 650 trees from AR samples (GL5221 and GL23596; 0.31%; *P* = .05, χ^2^ test) but none in the 1,094 trees from nonallergic samples.

**Table I:** Lineage tree frequency with clonal relatedness between IgE and other classes

IgE related to:	NA group	AR.IS and AR.OS groups
IgM	3∗ (0.29%)†	2 (0.31%)
IgA and IgM	1 (0.10%)	0
IgA	2 (0.19%)	2 (0.31%)
IgG	0	1 (0.15%)
IgG and IgM	0	1 (0.15%)

∗ Absolute number observed.

† Frequency is calculated based on clone counts in Table E3.

## Discussion

Previous studies of the human IgE repertoire were restricted by the use of low-throughput Sanger sequencing. To the best of our knowledge, the largest number of IgE sequences analyzed in a single report was limited to 1,366 IgE sequences isolated from the blood of 13 asthmatic children.^9^ Here we applied a high-throughput NGS approach and captured 8,135 IgE sequences (525 IgE clonotypes) from the peripheral blood and nasal mucosa of 9 adults. The small number of subjects reported here means that we are unable to draw general conclusions regarding the development of IgE repertoires in allergic disease. This would require a much larger cohort of patients or multiple types of studies with different patient groups to account for interindividual variations that naturally arise from differing conditions of sensitization and many clinical phenotypes (including disease severity and polymorphisms within the immunoglobulin gene locus). Despite these limitations, we have demonstrated the value of NGS repertoire analysis in allergy research and have been able to assess the influence of natural pollen exposure on the *IGH* repertoire in a small cohort of patients with AR.

Antigen stimulation often leads to selection for or against particular *IGH* gene rearrangements, resulting in altered repertoire diversity and abundance of immunoglobulin genes. In this report we observed a more limited diversity for IgE clones compared with other antibody classes, indicating that IgE clones might be more stringently selected. Furthermore, in contrast to a previous report describing similar use of *IGHV* between IgM and IgE repertoires,^9^ the IgE clonotypic repertoires reported here had characteristically higher use of *IGHV1* and *IGHV5* gene families but lower *IGHV3* use independent of atopic status compared with IgM. Although our sequence data alone cannot determine the nature of antigen stimulation, the distinct repertoire profile of IgE clonotypes suggests that IgE^+^ B cells might be subject to selection pressures different from other antibody classes. The overabundance of the minor *IGHV5* subgroup in the IgE repertoire has been suggested to associate with the pathogenesis of allergic disease,^6^, ^8^, ^12^ as previously demonstrated by comparing IgE and IgM repertoires within allergic subjects.^6^, ^7^ However, our results suggest that the overabundance of *IGHV5* might be specific to IgE but not to allergy *per se*. Further studies are required to confirm this finding and exclude any confounding variables related to disease status or sampling efficiency.

Recent data from a mouse model suggest that high-affinity IgE antibodies might arise through high-affinity, hypermutated IgG intermediates that undergo sequential CSR to IgE.^37^ Although previous studies showed that IgE transcripts in patients with AR and asthma are heavily mutated,^9^, ^10^ the effect of allergen exposure on GC reactions has not hitherto been directly demonstrated. Here we have shown that IgE sequences were highly mutated, regardless of atopic status. Crucially, our results demonstrated that IgE sequences from patients with AR were even more mutated and more varied during the pollen season. One potential explanation for these observations is that hypermutation is accumulated at a faster rate for IgE^+^ B cells in allergic patients under the influence of allergen exposure. Alternatively, increased mutations for IgE transcripts in season could be associated with enhanced sequential CSR from IgG to IgE, as previously reported in asthmatic patients.^38^ To test this, we performed a lineage tree analysis, which was mainly used to determine clonal relatedness across antibody classes but not to delineate the exact developmental progression *in vivo*, given the unlikeliness of capturing all clonal members at the time of sampling. For example, it would be biologically implausible for IgG_2/4_ to switch to IgG_1/3_, as illustrated in our own lineage trees.^39^ In such cases the hierarchic relationships suggest the existence of a common precursor rather than direct descent. With this caveat, the clonal relatedness we observed between IgE and IgG in samples from allergic patients, in contrast to its absence in healthy control subjects, suggests that sequential switching through IgG to IgE is associated with allergic disease. This prediction is amenable to further investigation with a larger cohort of patients. Nevertheless, it is supported by the increased frequency of Iɛ-Cγ switch circle transcripts (transient markers of CSR from IgG to IgE) in the bronchial mucosa of asthmatic patients compared with healthy control subjects.^38^

Hypermutation patterns have detrimental effects on the selection of B cells and, subsequently, the diversity and affinity of antibodies. A previous study showed evidence of negative selection in framework regions but did not observe any specific patterns for CDRs in IgE sequences.^40^ By using BASELINe to quantify selection strength, we detected significant evidence of antigen-driven selection for IgE transcripts, particularly in peripheral blood. Furthermore, our data demonstrate a unique selective environment that operates among patients with AR in season and differing pressures between peripheral blood and nasal mucosal compartments. Specifically, we observed that although IgE clones in peripheral blood taken in season were more negatively selected and subsequently had reduced clonal diversity, IgE clones in nasal mucosae taken in season were more positively selected, contributing to an increase in their clonal diversity.

It remains unclear exactly where, if at all, IgE^+^ B cells undergo affinity maturation. Increasing evidence suggests that GC-like structures containing IgE^+^ B cells are present in both the upper and lower airways in patients with AR and allergic asthma, respectively.^38^, ^41^, ^42^ Here we observed evidence of clonal expansion and increases in *IGHV* mutations and diversity of IgE clones isolated from the nasal mucosa. These observations were consistent with stronger positive selection that we observed in mucosal IgE in patients with AR in season. In part, these results might reflect selection of IgG clones and formation of IgG^+^ memory cells in the primary sensitization to allergens localized in the target organ, followed by allergen stimulation of clonal expansions and CSR to IgE. Using lineage tree analysis, we also found molecular footprints that linked IgE to other antibody classes in the nasal biopsy specimens, supporting this hypothesis. Altogether, our observations point to the possibility that the allergic respiratory mucosa supports local affinity maturation of IgE, which is consistent with the detection of activation-induced cytidine deaminase and switch circle transcripts in the target organ in patients with AR and asthma, as well as nasal polyposis.^38^, ^42^, ^43^ However, it is entirely possible that some IgE transcripts detected in nasal biopsy specimens could represent plasma cells that have undergone GC reactions in regional lymph nodes before migrating to the nasal mucosa.^44^ Further studies on cell trafficking in the human system will help to address both possibilities.

There are important questions that remain to be answered. First, as previously reported for atopic dermatitis,^11^ we observed a fraction of germline-like IgE transcripts in all groups independent of atopic status. Presumably, this germline-like IgE^+^ population is derived through direct CSR from IgM to IgE and has been suggested to arise through polyclonal activation of B1-like CD5^+^ B cells.^11^ Because the relative proportion of germline-like versus mutated IgE^+^ populations was different between allergic and healthy subjects, directly characterizing these IgE^+^ populations both phenotypically and functionally might help us better understand their differing roles in allergic responses.

Second, we observed that IgE sequences taken in season from patients with AR were more mutated but had longer CDR-H3. Increased hypermutation and shorter CDR-H3 are suggestive of specific antigen-driven responses, whereas polyreactivity has been associated with longer CDR-H3.^35^, ^36^ The true relevance of the increased CDR-H3 lengths we have observed in IgE sequences from allergic patients remains to be determined, which will require generation of recombinant antibodies for functional analysis.^11^, ^45^

Third, whether the alterations observed in non-IgE repertoires are relevant to the development of IgE^+^ B cells and the nature of allergic response in patients with AR remains to be investigated in future studies.

In conclusion, we observe that the pattern of *IGH* gene rearrangements in the IgE repertoire was similar for all groups but distinct from other antibody classes independent of atopic status. Our data demonstrate seasonal and compartmental differences in clonal diversity and selection strength of IgE and provide direct evidence of increased hypermutation for IgE^+^ cells in patients with AR under the influence of natural pollen exposure. Our lineage tree analysis reveals intraclonal diversification of IgE clones and association of IgE repertoires between the blood and nasal mucosal compartments. Finally, clones shared between IgE and IgG classes are detected in allergic subjects but absent in nonallergic subjects, which is indicative of preferential sequential switching through IgG in allergic subjects. Taken together, these data demonstrate NGS as a powerful approach to study the immune repertoire in patients with allergic diseases.

**Key messages**

• Natural exposure to grass pollen is associated with enhanced SHM, increased diversity, and changes in selection and CSR patterns for IgE repertoires in patients with AR.
• IgE repertoires linking peripheral blood and nasal mucosal compartments have a distinct pattern of *IGH* rearrangements independent of atopic status.
• We demonstrate the technical feasibility and analytic power of NGS for the determination of immunoglobulin repertoires in patients with respiratory allergic disease.

## Figures and Tables

**Figure:**
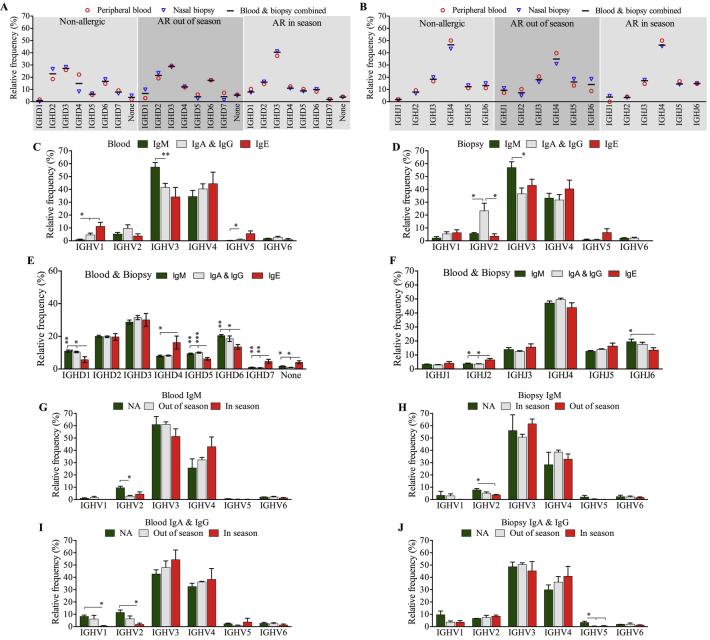
Fig E1

**Figure:**
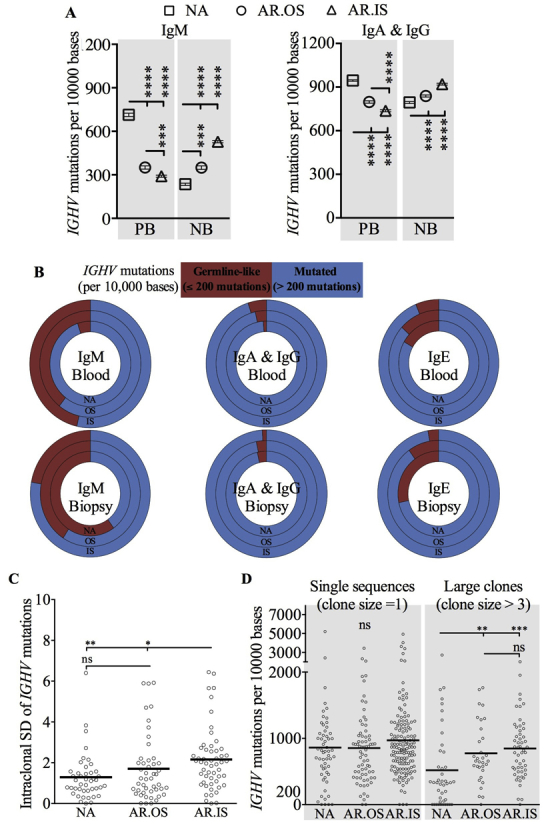
Fig E2

**Figure:**
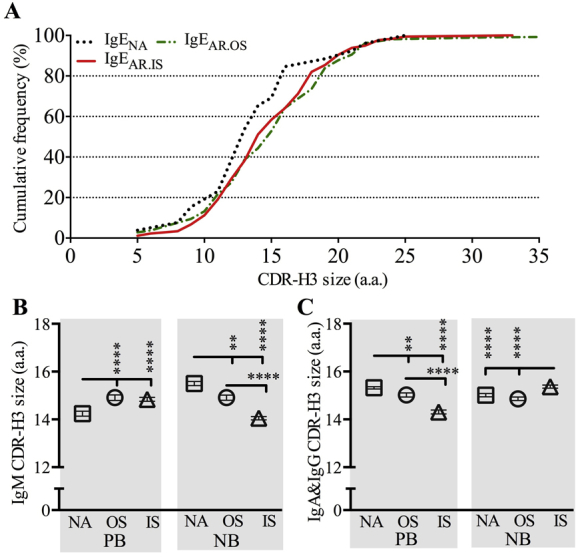
Fig E3

**Figure:**
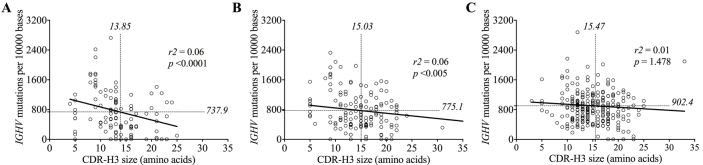
Fig E4

**Figure:**
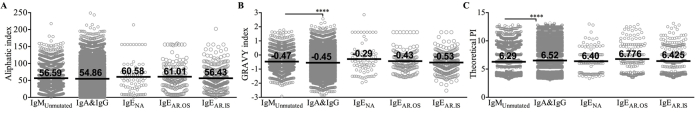
Fig E5

**Figure:**
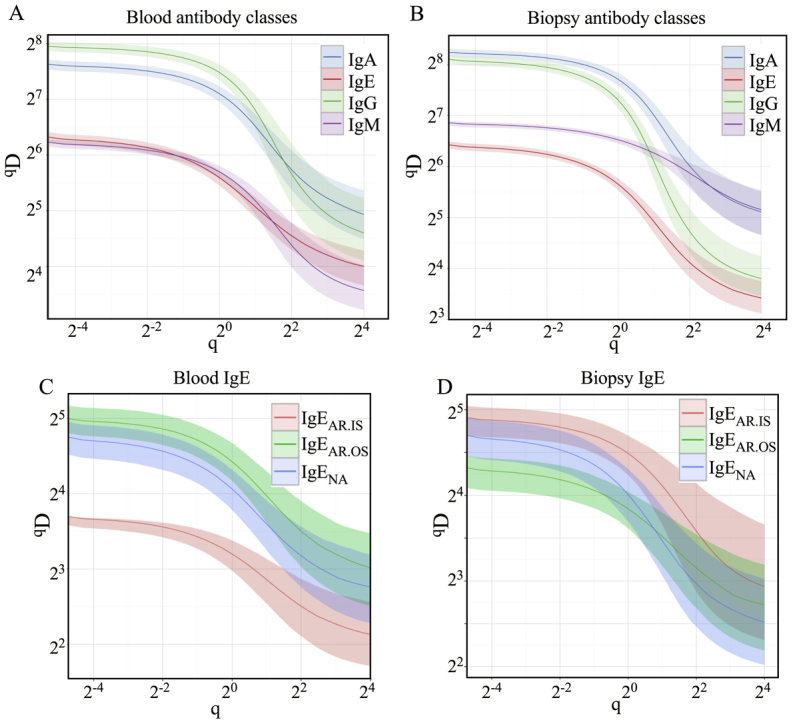
Fig E6

## Supplementary data

Online Repository Data

Online Repository References

Online Tables E1-E6

Figure Legends E1-E6
