# Supplementary material for: Influence of seasonal exposure to grass pollen on local and peripheral blood IgE repertoires in patients with allergic rhinitis
Source: J Allergy Clin Immunol. 2014 Sep;134(3):604–12. doi: 10.1016/j.jaci.2014.07.010 (PMC4151999; doi:10.1016/j.jaci.2014.07.010)
Supplement: Online Repository Data [file mmc1.doc]

**Title: Influence of allergen exposure on local and peripheral IgE repertoires in rhinitis**

**Online Repository Materials**

Additional Online Repository Materials include

Data E1. Method: Generation of *IGH* libraries

Data E2. Method: Sequence analysis pipeline

Data E3. Method: Stringent clonotype clustering

Data E4. Method: Quantifying selection strength in IgE sequences using BASELINe

Data E5. Method: *IGH* repertoire diversity using Hill’s model

Data E6. Method: Statistical analysis

Table E1. Characteristics of study participants

Table E2. PCR primers and sequences

Table E3. Numbers of *IGH* sequences

Table E4. Numbers of *IGH* clonotypic sequences

Table E5. Number of lineage trees in NA and AR samples

Table E6. Sequence information for clones containing IgE and related variants of other antibody classes

Figure E1. *IGH* gene usage in clonotypic sequences by antibody class and sample type

Figure E2. Mutation patterns in different samples and in expanded clones

Figure E3. Patterns of CDR-H3 size by antibody class and sample type

Figure E4. Correlative relationships between *IGHV* mutation and CDR-H3 size of IgE sequences

Figure E5. CDR-H3 peptide characteristics.

Figure E6. Detailed *IGH* repertoire diversity using Hill’s model Online Repository Materials

**Data E1 Method: generation of *IGH* libraries**

*IGH* genes were amplified from cDNA in semi-nested PCR reactions, with minor changes of previously reported methods (E1). In brief, PCR1 reaction mix (20μl) consisted of 2 μl cDNA, 200 μM each dNTP (Promega, UK), 0.4U Phusion DNA polymerase (NEB, UK), six FW1/*IGHV* gene family primers (41.75 nM each) in conjunction with constant region primers (either IGα-1, IGγ-1, IGε-1, or IGμ-1; 250 nM; Table E2). Subsequently, re-amplification from 2.5 μl PCR1 products were performed in PCR2 reaction mix (25 μl), comprising 200 μM each dNTP, 0.5 U Phusion DNA polymerase and six FW1/*IGHV* gene family primers (41.75 nM each) in combination with antibody class specific, nested constant region primers (IGα-2, IGγ-2, IGε-2, or IGμ-2; 250 nM) where all primers were labeled at the 5’-end with Multiplex Identifiers (MIDs) for multiplexing of twelve different libraries. PCR thermal cycling is as follows: 98°C for (30 s), 25(PCR1), or (PCR2) cycles of 98°C (10 s); 60°C (15 s); 72°C (45 s), and 1 cycle of 72°C (5 min). To produce sufficient amounts of amplicons for pyrosequencing on the 454 GS FLX+ System, eight different PCR1 reactions per sample, followed by two PCR2 reactions (16 reactions in total per sample) were performed for each class, which were then purified from 1.5% TAE agarose gels using the Silica Bead Gel Purification Kit (Thermo Scientific, UK). Amplicons from 16 reactions and four classes were combined as one library (using the same MID barcode), and further enriched using the QIAquick PCR Purification Kit (Qiagen, UK).

**Data E2 Method: Sequence analysis pipeline**

This was performed as previously described (E2) but with extensions to include IgE sequences. In the initial quality control step a range of criteria are used to identify and then exclude artefactual sequences that may have arisen as a consequence of PCR or Online Repository Materials Wu *et al.* 3 ligation errors, as well as a more general requirement to meet various markers of biological plausibility based on known information about the germline sequence.

Sequences that pass quality control are then annotated using IMGT/HighV-QUEST (E3) and the output files parsed into a single table. The nucleotide sequences for the defined CDR-H3 regions were then used to compare all sequences in a pairwise fashion. For each pairwise comparison the minimum nucleotide edit distance (including indels) between CDR-H3s was calculated and standardised to the shorter of the two CDR-H3 regions. In those cases where the standardised edit distance was greater than 0.2, or where *IGHV* gene identity was called for both sequences by IMGT HighV-QUEST and those *IGHV* genes did not match, then the edit distance was set to 1. This forces those two sequences to be in separate clusters, and also ensures that all members of a cluster have the same *IGHV* gene. This distance matrix was then subjected to hierarchical clustering (complete linkage) and the subsequent dendrogram was cut at a defined height so as to generate the final clusters (clones). Finally, for each cluster an exemplar sequence was chosen to represent that clone in downstream analysis. These were determined predominantly on the basis of being the modal sequence in the cluster, but also with some weight given to *IGHJ* family usage (so as to generate an exemplar sequence that illustrated the most common sequence and *IGHJ* family usage). All analyses before and after IMGT/HighV-Quest were performed in R (E4) using custom scripts. The background error frequency for the whole system was approximately 2% (E2).

**Data E3. Method: Stringent clonotype clustering**

Prior to analysis of selection strength, diversity and lineage, more stringent clustering criteria were imposed on QC-filtered sequence data. First, an initial "preclone" categorical grouping required sequences to share the same clonotypic characteristics (the same *IGHV* genes, *IGHJ* genes and CDR-H3 junction length). A second step that further divided "preclones" into clones based on a within-clone junctional nucleotide distance of 5. Distance was measured as the number of point mutations weighted by a symmetric version of the nucleotide substitution probability previously described (E5).

**Data E4. Method: Quantifying selection strength in IgE sequences using BASELINe**

Selection strength was estimated as previously described (E6) with the Focused test statistic and S5F mutation model (E7). In brief, BASELINe quantifies selection strength (Σ) as the log-odds ratio between the observed and expected replacement frequencies. The expected frequencies are calculated using the S5F model to account for SHM hot/cold-spots and substitution preferences. Rather than a single point estimate for the selection strength, BASELINe utilizes a Bayesian framework to compute the complete probability density for Σ, thus providing a quantification of uncertainty in the analysis. Positive Σ values indicate an increased frequency of replacements (positive selection), while negative Σ values indicate a decreased frequency (negative selection) relative to a background model of SHM.

**Data E5. Method: *IGH* repertoire diversity using Hill’s model**

The repertoire diversity was analysed using the method proposed by Hill (E8). This method quantifies diversity as a smooth function (qD) of a single parameter (*q*):

where S is the number of clones, pi is the proportional abundance of the ith clone and the parameter q determines how clonal frequencies are weighted. Special cases of this general index of diversity correspond to the most popular diversity measures in ecology: species richness (q = 0), the exponential Shannon–Weiner index (q = 1), and the inverse of the Simpson index (q = 2).

At q=0 different species weight equally, regardless of their abundance or rarity within the population and this total clonal diversity (0D) is illustrated in Figure 5. In general, as the parameter *q* varies from 0 to +∞ the diversity number (qD) depends less on rare species and more on common (abundant) ones, thus encompassing a range of definitions that can be visualized as a single curve (Fig E6). For example in Figure E6, *A*, as q values increased, the IgE diversity was similar to that of IgG and IgM in the blood (i.e. it was no more dominated by a single clone). In contrast, IgA in the blood tended to be more dominated by a single clone. This full curve contains significantly more information than any single measure, and is thus preferable to the approaches used in most existing studies. To account for differences in sequencing depth, the number of sequences in each diversity calculation was set to the minimum across groups through repeated sub--‐sampling with replacement. Confidence intervals were constructed using the percentile method and a 95% confidence interval for each qD was determined via bootstrapping (2,000 permutations).

**Data E6. Method: Statistical analysis**

Different statistical approaches were used to compare groups in each figure.

Figure 1, *A*; Figure E1, *A* & *B*: χ*2* test with Sidak adjustment using the absolute count of clonotypes by *IGHV* family usage pooled from multiple individuals.

Figure 1, *B* & *C*; Figure E1, *C-F*: RM one-way ANOVA with the Greenhouse-Geisser correct (parametric, paired) with Holm-Sidak’s multiple comparisons test using the relative frequency derived from the absolute count of clonotypes by gene usage within individuals for comparison between antibody classes.

Figure E1, *G*-*J*: Ordinary one-way ANOVA (parametric, unpaired) with Holm-Sidak’s multiple comparisons tests using the relative frequency derived from the absolute count of clonotypes by gene usage within individuals for comparison between antibody classes.

Figure 2, *A* & *B*; Figure 3, *B*-*D*; Figure E2, *A*, *B, D, E*; Figure E3 *B* & *C;* Figure E5: Kruskal- Wallis test (non-parametric, unpaired), with Dunn's post-test for multiple comparisons.

Figure 2, *C* - *F*: Mann-Whitney test (non-parametric, unpaired).

Figure 5: P-values for comparing the diversities of two groups (*a* and *b*) were generated via bootstrap of Δ0*D,* the difference between the estimated diversity in each group. Each bootstrap realization *i* is generated by resampling (with replacement) equivalent numbers of sequences from groups a and *b*, and calculating:

where B=2,000 is the number of bootstrap realizations, and

is the diversity score at *q*=0 (Data 5) for group *g*. For each comparison, group *a* is defined by having the higher median diversity compared with group *b*. The p-value is then calculated using the percentile method and doubled to account for the two-tailed nature of the test.

Figure 6: Focused test statistic and S5F mutation model.
