# Supplementary material for: Influence of seasonal exposure to grass pollen on local and peripheral blood IgE repertoires in patients with allergic rhinitis
Source: J Allergy Clin Immunol. 2014 Sep;134(3):604–12. doi: 10.1016/j.jaci.2014.07.010 (PMC4151999; doi:10.1016/j.jaci.2014.07.010)
Supplement: Online Repository References [file mmc2.doc]

**References**

E1. Wu YC, Kipling D, Leong HS, Martin V, Ademokun AA, Dunn-Walters DK. High--throughput immunoglobulin repertoire analysis distinguishes between human IgM memory and switched memory B-cell populations. Blood. 2010;116(7):1070-8.

E2. Ademokun A, Wu YC, Martin V, Mitra R, Sack U, Baxendale H, et al. Vaccination-induced changes in human B-cell repertoire and pneumococcal IgM and IgA antibody at different ages. Aging Cell. 2011;10(6):922-30.

E3. Alamyar E, Duroux P, Lefranc MP, Giudicelli V. IMGT((R)) tools for the nucleotide analysis of immunoglobulin (IG) and T cell receptor (TR) V-(D)- J repertoires, polymorphisms, and IG mutations: IMGT/V-QUEST and IMGT/HighV-QUEST for NGS. Methods Mol Biol. 2012;882:569-604.

E4. Team RDC. R: A language and environment for statistical computing. R Foundation for Statistical Computing, Vienna, Austria. ISBN 3-900051-07-0. 2011.

E5. Smith D, Creadon G, Jena P, Portanova J, Kotzin B, Wysocki L. Di- and trinucleotide target preferences of somatic mutagenesis in normal and autoreactive B cells. J. Immunol. 1996;156:2642-52.

E6. Yaari G, Uduman M, Kleinstein SH. Quantifying selection in high-throughput Immunoglobulin sequencing data sets. Nucleic Acids Res. 2012;40(17):e134.

E7. Yaari G, Vander Heiden JA, Uduman M, Gadala-Maria D, Gupta N, Stern JN, O'Connor KC, Hafler DA, Laserson U, Vigneault F, Kleinstein SH. Models of somatic hypermutation targeting and substitution based on synonymous mutations from high- throughput immunoglobulin sequencing data. Front Immunol. 2013;4:358. doi: 10.3389/fimmu.2013.00358.

E8. Hill MO. Diversity and Evenness: A Unifying Notation and Its Consequences. Ecology. 1973;54(2):427-32.
