# Supplementary material for: Influence of seasonal exposure to grass pollen on local and peripheral blood IgE repertoires in patients with allergic rhinitis
Source: J Allergy Clin Immunol. 2014 Sep;134(3):604–12. doi: 10.1016/j.jaci.2014.07.010 (PMC4151999; doi:10.1016/j.jaci.2014.07.010)
Supplement: Online Tables E1-E6 [file mmc3.docx]

**Title: Influence of allergen exposure on local and peripheral IgE repertoires** **in rhinitis**

**Tables for Online Repository Materials**

**Table E1. Characteristics of study participants^1^.**

| **ID** | **301^2^** | **302** | **303** | **304** | **310** | **311** | **306** | **305** | **307** | **308** |
| --- | --- | --- | --- | --- | --- | --- | --- | --- | --- | --- |
| **Group** | AR. IS | AR. IS | AR. IS | AR. IS | AR. OS | AR. OS | AR.  OS | NA | NA | NA |
| **Sample Month** | July | July | July | Aug | Jan | Jan | Dec | Dec | Dec | Dec |
| **Age (years)** | 27 | 43 | 28 | 20 | 33 | 41 | 27 | 31 | 54 | 28 |
| **Sex** | F | F | F | F | F | F | F | F | M | F |
| **SPT^3^** | TG,  SB, C, HD, AA,  CH | TG, SB,  C, D,  HD,  AA | TG,  HD, C, D | TG,  SB,  D, C, HDM | TG,  AA,  D, C, HDM | TG, D, C, HDM | TG, SB, C,  HDM,  AA, CH | -ve | -ve | -ve |
| **Total IgE (kU/L)** | 261 | 556 | 457 | 128 | 1690 | 17 | 261 | 13 | 6 | nd |
| **Timothy-grass  specific IgE (kU/L)** | 56.9 | 40 | 35.2 | 16.7 | 0.08 | 0.58 | 56.9 | <0.01 | <0.01 | nd |
| **SRA**  **accessions^4^** | SRX472476 | SRX472474 | SRX472473 | SRX472471 | SRX472417 | SRX472470 | SRX472466 | SRX472468 | SRX472478 | SRX472477 |

**^1^**Allergic status was assessed on the basis of medical history or skin prick tests and serum total and allergen-specific IgE. None of the participants had previously received allergen immunotherapy or had any other significant nasal or systemic disease.
**^2^**Same individual.
**^3^**TG: Timothy Grass; SB: Silver Birch; C: Cat; HDM: House Dust Mite; AA: Altemaria alternata; CH: Cockroach; H: Horse; D: Dog; nd, not done.
^4^All QC-filtered sequences (Table E3) are accessible on Sequence Rear Archive (SRA), NCBI (http://www.ncbi.nlm.nih.gov/Traces/sra/sra.cgi?view=studies) using the SRA study accession number SRP038092. Within SRP038092, sequences from each subject can be identified by SRX accessions.

**Table E2. PCR primers and sequences^1^.**

| **PCR1 primers** | | | | |
| --- | --- | --- | --- | --- |
| Primer names | | Sequences (5’ to 3’) | | |
| FW1/IGHV1/7 | | CCTCAGTGAAGGTCTCCTGCAAGG | | |
| FW1/IGHV2 | | TCCTGCGCTGGTGAAACCCACACA | | |
| FW1/IGHV3 | | GGTCCCTGAGACTCTCCTGTGCA | | |
| FW1/IGHV4 | | TCGGAGACCCTGTCCCTCACCTGC | | |
| FW1/IGHV5 | | CAGTCTGGAGCAGAGGTGAAA | | |
| FW1/IGHV6 | | CCTGTGCCATCTCCGGGGACAGTG | | |
| IGα-1 | | GGCTCCTGGGGGAAGAAGCC | | |
| IGγ-1 | | GCGCCTGAGTTCCACGACAC | | |
| IGμ-1 | | GGGGAATTCTCACAGGAGAC | | |
| IGε-1 | | TGTGTCGCAGGTCACCATCACGC | | |
| **PCR2 primers** | | | | |
| **Multiplex identifiers** | | | **PCR2 gene-specific sequences** | |
| Barcodes | Sequences  (5’ to 3’) | | Primer names | Sequences (5’ to 3’) |
| MID 1 | acgagtgcgt | | FW1/IGHV1/7 | CCTCAGTGAAGGTCTCCTGCAAGG |
| MID 2 | acgctcgaca | | FW1/IGHV2 | TCCTGCGCTGGTGAAACCCACACA |
| MID 3 | agacgcactc | | FW1/IGHV3 | GGTCCCTGAGACTCTCCTGTGCA |
| MID 4 | agcactgtag | | FW1/IGHV4 | TCGGAGACCCTGTCCCTCACCTGC |
| MID 5 | atcagacacg | | FW1/IGHV5 | CAGTCTGGAGCAGAGGTGAAA |
| MID 6 | atatcgcgag | | FW1/IGHV6 | CCTGTGCCATCTCCGGGGACAGTG |
| MID 7 | cgtgtctcta | | IGα-2 | GGAAGAAGCCCTGGACCAGGC |
| MID 8 | ctcgcgtgtc | | IGγ-2 | CACCGTCACCGGTTCGGGG |
| MID 9 | tagtatcagc | | IGμ-2 | CAGGAGACGAGGGGGAAAAGG |
| MID 10 | tctctatgcg | | IGε-2 | CGGAGGTGGCATTGGAGGG |
| MID 11 | tgatacgtct | | Full-length PCR2 primers contain MID sequence attached to 5’-end of gene-specific sequences. | |
| MID 12 | tactgagcta | |  |  |
| **Examples of PCR2 MID primers** | | | | |
| **Primer names** | | | **Sequences (5’ to 3’)** | |
| MID1: FW1/IGHV3 | | | acgagtgcgtGGTCCCTGAGACTCTCCTGTGCA | |
| MID3: IGε-2 | | | agacgcactcCGGAGGTGGCATTGGAGGG | |
| MID3: IGε-2 | | | agacgcactcCGGAGGTGGCATTGGAGGG | |

^1.^All except for IgE constant region primers were as previously described (E1).

**Table E3. Number^1^ of *IGH* sequences.**

| **Status** | **Samples** | **IgM** | **IgA** | **IgG** | **IgE** | **Unknown class^2^** | **Total** |
| --- | --- | --- | --- | --- | --- | --- | --- |
| Non-allergic  (NA; n=3) | PB | 2853 | 9545 | 10570 | 2351 | 1967 | 27286 |
|  | NB | 2989 | 8230 | 8562 | 1544 | 2095 | 23420 |
| AR out of season (AR._OS_; n=3) | PB | 1319 | 2142 | 1974 | 2569 | 930 | 8934 |
|  | NB | 1568 | 3059 | 2435 | 389 | 1366 | 8817 |
| AR in season  (AR._IS_; n=4) | PB | 2240 | 1916 | 2810 | 353 | 1480 | 8799 |
|  | NB | 5197 | 4872 | 6768 | 929 | 2588 | 20354 |
| **Total** | | 16166 | 29764 | 33119 | 8135 | 10426 | **97610** |

**^1^**Only QC-filtered sequences with identifiable CDR-H3 are included and the total number of sequences from multiple subjects (n) is indicated. **^2^**Unknown class refers to sequences that had complete VDJ rearrangements but did not extend into the constant region for the identification of antibody classes.

**Table E4. Number of *IGH* clonotypic^1^ sequences.**

| **Status** | **Samples** | **IgM** | **IgA** | **IgG** | **IgE** | **Total** |
| --- | --- | --- | --- | --- | --- | --- |
| Non-allergic  (NA; n=3) | PB | 1292 | 4379 | 2978 | 54 | 8703 |
|  | NB | 2232 | 2414 | 1729 | 60 | 6435 |
| AR out of season  (AR._OS_; n=3) | PB | 1089 | 1280 | 1046 | 68 | 3483 |
|  | NB | 1175 | 1713 | 1174 | 81 | 4143 |
| AR in season  (AR._OS_; n=4) | PB | 1954 | 1139 | 1472 | 48 | 4613 |
|  | NB | 3033 | 2658 | 1893 | 214 | 7798 |
| **Total** | | 10775 | 13583 | 10292 | 525 | **35175** |

**^1^**Only one sequence, i.e. clonotypic sequence, was chosen to represent each clonotype. Clonotypes with unknown classes are not shown.

**Table E5. Number of lineage trees in NA and AR samples^1^.**

| **Class^2^** | **NA** | | | **AR** | | | **Grand total** |
| --- | --- | --- | --- | --- | --- | --- | --- |
|  | PB**^3^** | PB/NB | NB**^4^** | PB^2^ | PB/NB | NB |  |
| A | 151 | 23 | 222 | 58 | 7 | 128 | 589 |
| A/E | 1 | 1 | 0 | 0 | 2 | 0 | 4 |
| A/E/M | 0 | 1 | 0 | 0 | 0 | 0 | 1 |
| A/G | 22 | 8 | 25 | 16 | 2 | 14 | 87 |
| A/G/M | 3 | 0 | 0 | 0 | 0 | 3 | 6 |
| A/M | 4 | 2 | 4 | 2 | 0 | 5 | 17 |
| E | 25 | 5 | 21 | 34 | 10 | 39 | 134 |
| E/G | 0 | 0 | 0 | 0 | 0 | 1 | 1 |
| E/G/M | 0 | 0 | 0 | 0 | 1 | 0 | 1 |
| E/M | 1 | 1 | 1 | 1 | 0 | 1 | 5 |
| G | 280 | 22 | 194 | 81 | 4 | 161 | 742 |
| G/M | 1 | 1 | 0 | 2 | 1 | 2 | 7 |
| M | 52 | 1 | 22 | 3 | 1 | 71 | 150 |
| **Grand total Total** | 540 | 65 | 490 | 198 | 28 | 425 | **1744** |
|  | 1094  65  490 | | | 650  28  426 | | |  |

**^1^**Lineage trees contain related variants with unique mutation patterns. **^2^**antibody classes (A: IgA, G: IgG, E: IgE and M: IgM); **^3^**PB: peripheral blood; **^4^**NB: Nasal biopsy

**Table E6. Sequence information for clones containing IgE and related variants of other antibody classes.**

| **Tree ID** | **Antibody classes** | **Patient** | **Unique/total^1^ sequences** | **IGHV** | **IGHJ** | **IGHD** | **CDR-H3 amino acids^2^** | **SAR accession^3^** |
| --- | --- | --- | --- | --- | --- | --- | --- | --- |
| GL22489 | IgA/IgE | 305 | 11/12 | IGHV4-39 | IGHJ3 | IGHD3-22 | AAWLFGLRSFDI | SRX485054 |
| GL4124 | IgA/IgE | 308 | 4/5 | IGHV4-59 | IGHJ6 | IGHD3-10 | SRGVVWFGDPLGITYNDYYYMDV | SRX485057 |
| GL34970 | IgA/IgE | 311 | 11/201 | IGHV4-4 | IGHJ4 | IGHD2-15 | ARDVAAFDS | SRX485051 |
| GL41187 | IgA/IgE | 311 | 8/151 | IGHV3-74 | IGHJ2 | IGHD7-27 | ASWVL | SRX485052 |
| GL18412 | IgA/IgE/IgM | 308 | 33/580 | IGHV1-2 | IGHJ5 | IGHD6-13 | ARAGPGIAAIGNWLDP | SRX485058 |
| GL5221 | IgE/IgG | 311 | 26/46 | IGHV2-5 | IGHJ6 | IGHD2-15 | AHLRSGYYAMDV | SRX485053 |
| GL23596 | IgE/IgG/IgM | 311 | 27/189 | IGHV4-39 | IGHJ4 | IGHD4-23 | ARHRWEGTWSFDY | SRX485063 |
| GL25649 | IgE/IgM | 303 | 6/6 | IGHV3-74 | IGHJ5 | IGHD7-27 | ARDFIEDCCPNNWFDP | SRX485061 |
| GL12091 | IgE/IgM | 305 | 14/259 | IGHV1-69 | IGHJ6 | IGHD6-13 | ARGPAAGIYGMDV | SRX485056 |
| GL39602 | IgE/IgM | 308 | 15/34 | IGHV4-39 | IGHJ3 | IGHD3-16 | ARHAETYYDYVWAPGVSAFDI | SRX485059 |
| GL39613 | IgE/IgM | 308 | 29/198 | IGHV4-39 | IGHJ3 | IGHD3-16 | ARHAETYYDYVWAPGGSAFDI | SRX485060 |
| GL583 | IgE/IgM | 311 | 7/11 | IGHV4-39 | IGHJ3 | IGHD2-2 | ARHRWEGTWSFD | SRX481895 |

**^1^**Sequence counts include duplicates.
**^2^**CDR-H3 amino acid motifs: the conserved first (cysteine) and last (tryptophan) amino acid determined by IMGT/HighV-Quest are now shown.

**^3^**Sequences of clonal members are accessible on Sequence Rear Archive (SRA), NCBI (http://www.ncbi.nlm.nih.gov/Traces/sra/sra.cgi?view=studies) using the SRA study accession number SRP038092. Within SRP038092, sequences from each subject can be identified by SRX accessions.
