# Supplementary material for: Influence of seasonal exposure to grass pollen on local and peripheral blood IgE repertoires in patients with allergic rhinitis
Source: J Allergy Clin Immunol. 2014 Sep;134(3):604–12. doi: 10.1016/j.jaci.2014.07.010 (PMC4151999; doi:10.1016/j.jaci.2014.07.010)
Supplement: Figure Legends E1-E6 [file mmc4.doc]

**Figure Legends**

**Figure E1. *IGH* usage of clonotypic repertoires by antibody class and sample type.** The use of **(A)** *IGHD* families and **(B)** *IGHJ* families in IgE clonotypic repertoires was compared across atopic status and sample types. The frequencies of *IGHV* families were compared between IgM, IgA & IgG and IgE repertoires **(C)** in the blood or **(D)** in the nasal biopsies. Comparisons for the usage of **(E)** *IGHD* families and **(F)** *IGHJ* families between antibody classes were performed using pooled blood and biopsy clonotypic sequences. The frequencies of *IGHV* families for **(G)** IgM in the blood, **(H)** IgM in nasal biopsies, **(I)** IgA & IgG in the blood and **(J)** IgA & IgG in nasal biopsies were compared between atopic status. **p*<.05; ***p*<.005.

**Figure E2. Mutation patterns in different samples and in expanded clones. (A)**  Mean mutation frequencies for IgM and IgA & IgG clonotypic sequences in the peripheral blood (PB) or nasal biopsy (NB) were compared between atopic status. **(B)** Pie charts show relative fractions of IgM and IgA & IgG clonotypes ranked by mutations in the blood and nasal biopsies from NA (inner circles), AR.OS (middle circles) and AR.IS (outer circles). **(C)** Clonal relatives were included and intraclonal SD of *IGHV* mutations for large IgE clones (clone size >3) was compared between atopic status. **(D)** Mean mutation frequencies for single IgE sequences or large IgE clones were compared between different atopic status. **p*<.05; ***p*<.005; ****p*<.0005; *****p*<.00005.

**Figure E3. Patterns of CDR--‐H3 size by antibody class and sample type. (A)** The cumulative frequency of clonotypic sequences with different CDR--‐H3 sizes is shown for IgENA (black), IgEAR.OS (green) and IgEAR.IS (red). Mean CDR--‐H3 size for **(B)** IgM and **(D)** IgA& IgG clonotypic sequences was compared between atopic status in the peripheral blood (PB) and nasal biopsies (NB). **p*<.05; ***p*<.005; *****p*<.00005.

**Figure E4. Correlative relationships between *IGHV* mutation and CDR--‐H3 size of IgE sequences.** Pearson correlation and linear regression was performed to determine the association between the *IGHV* mutation and CDR--‐H3 size for **(A)** IgENA, **(B)** IgEAR.OS and **(C)** IgEAR.IS clonotypic sequences. Dash lines indicate the mean values for *IGHV* mutations and CDR--‐H3 sizes.

**Figure E5. CDR--‐H3 peptide characteristics. (A)** Aliphatic index, **(C)** GRAVY index and  **(D)** Theoretical PI was compared between groups as indicated. *****p*<.00005.

**Figure E6. Detailed *IGH* repertoire diversity using Hill’s model** Diversity (qD) from q=0 to q=∞ was analysed using Hill’s model for **(A)** different antibody classes in the blood, **(B)** different antibody classes in nasal biopsies, **(C)** IgE in the blood and **(D)** IgE in nasal biopsies from different atopic status. Diversity scores at q=0 (0D) were extracted and shown in Figure 5. Median diversity (dark lines) and 95% confidence intervals (shaded bands) are indicated. Subsampling was set to the lowest unique sequence count: **(A)** 519 and **(B)** 723 sequences per antibody class; **(C)** 128 and  **(D)** 108 sequences per atopic status.
